# Electric Clocks for Hospitals

**Published:** 1911-03-25

**Authors:** 


### ELECTRIC CLOCKS FOR HOSPITALS.

As in every other large institution, if things are to go smoothly it is important not only to keep time, but that the time shall be the same in every part of the hospital. To meet this requirement, electrically controlled clocks have been gradually developed. It is now a long time since the idea of controlling clocks by electricity was introduced. As usual, a great many difficulties have had to be overcome. It is the same with all new developments. The problem usually seems very simple when it is first approached; but as experience is gained from the apparatus designed to meet the requirements, one difficulty after another arises which no one would have dreamed of, and in too many instances the apparatus is thrown on one side.

In addition to the advantage of having all the clocks about a hospital keeping exactly the same time, it is a great advantage not to be obliged to wind them. Where there are necessarily a large number of clocks, as in any hospital of any size, the winding of the clocks periodically takes up a certain time, and in the past has afforded employment to certain individuals. The hospital was more or less at the mercy of these individuals, not only for the actual winding of the clocks, but for winding them at the proper time. As everyone who has had anything to do with clocks knows, good timekeeping depends very largely upon winding the clocks at the proper time. If they are allowed to go over their time they are apt to lose.

With electrically controlled clocks all the winding that is done and all the control is quite automatic.

There is a master clock fixed in any convenient portion of the building, say, in the porter's lodge. It winds itself automatically by the aid of an electric current, and at certain definite intervals, usually every half-minute, a current is sent from the master clock, through an electro-magnetic device in each of the controlled clocks, by means of which the minute hand of the controlled clock is pushed forward. The minute hand as it moves round of course moves the hour hand with it, so that the whole of the clocks in the building move forward together. The arrangement of the master clock for performing the work that is usually done either by a weight or a spring varies, but the main lines are very much as follows. The master clock has a pendulum, swinging in the usual way, and provided with a light contact hanging beneath it. When the pendulum swings freely, covering its full arc, the light contact spade swings clear of an electrical contact device placed in its path below. When the pendulum has slowed very slightly, the light contact device makes connection between the two contact pieces below, and a current is sent through an electro-magnetic device in the clock, the electro-magnet automatically performing the office that would be performed in the ordinary way by the descent of a weight, or the uncoiling of a spring. The electrical method of keeping the clock going in this way has the great advantage that the impulse is always the same. As our readers know, the impulse of a descending weight is not always the same, nor of an uncoiling spring. Complicated devices have to be added to every clock to make up for the difference in the impulses imparted to the pendulum. In the electric clock the whole thing is maintained in a very simple form.

In addition to the above arrangement, Messrs. Gent and Co., of Faraday Works, Leicester, who have done a very large amount of work in connection with electrically driven and controlled clocks, have added to their system an arrangement by which the battery which furnishes the current gives warning some time before it requires renewal. Current may be used for the clocks taken from the ordinary electrical supply service of the town; but the more general practice is to employ a battery of dry cells similar to that used for electric bells and telephones. The employment of a battery renders the whole thing independent of any breakdowns in the town supply service. Batteries, however, as those who have electric bells and telephones know, work down after a time, and one of the difficulties which had to be met was knowing when to renew the battery. This, of course, can be arranged by periodical visits of skilled workmen to test the batteries; but this means expense, and again it places the users of clocks in the hands of the workman. The automatic device which gives warning by ringing a bell when the strength of the battery falls below a certain figure completely overcomes the difficulty. The warning is given in plenty of time to enable all that is required to be done to the battery. Messrs. Gent make control and controlled clocks for all purposes—for turrets, for wards, for the outside of buildings, and for pretty well every purpose. They advise us also that they make perfectly noiseless electrically controlled clocks for special cases where the tick of a clock or the sound of the automatic device is objectionable. They have also worked out one very beautiful apparatus, by the aid of large electric lights, or any electric current that requires to be turned on, say, during hours of darkness and turned off at daylight, or *vice versa*, is controlled automatically. The arrangement comprises a special form of clock movement, which there is not space to describe here. It has been worked out mathematically. The result is, electrical contacts are made at the right time on each day, notwithstanding the later hour of sunset and the earlier hour of sunrise during a portion of the year, and the earlier hour of sunset and the later hour of sunrise during the remainder of the year, day by day.

